# Assessment of *Polygala paniculata* (Polygalaceae) characteristics for evolutionary studies of legume–rhizobia symbiosis

**DOI:** 10.1007/s10265-019-01159-x

**Published:** 2019-12-11

**Authors:** Yuji Tokumoto, Kayo Hashimoto, Takashi Soyano, Seishiro Aoki, Wataru Iwasaki, Mai Fukuhara, Tomomi Nakagawa, Kazuhiko Saeki, Jun Yokoyama, Hironori Fujita, Masayoshi Kawaguchi

**Affiliations:** 1 Division of Symbiotic Systems, National Institute for Basic Biology, Okazaki, Aichi 444 8585 Japan; 2 Department of Basic Biology, School of Life Science, SOKENDAI (The Graduate University for Advanced Studies), Okazaki, Aichi 444 8585 Japan; 3 Department of Biological Sciences, Graduate School of Science, The University of Tokyo, Tokyo, 113 0032 Japan; 4 Division of Biological Science, Graduate School of Science, Nagoya University, Nagoya, Aichi 464 8602 Japan; 5 Department of Biological Sciences and Kyousei Science Center for Life and Nature, Nara Women’s University, Nara, 630 8506 Japan; 6 Faculty of Science, Yamagata University, Yamagata, 990 8560 Japan; 7 Astrobiology Center, Mitaka, Tokyo 181 8588 Japan; 8 Atmosphere and Ocean Research Institute, The University of Tokyo, Kashiwa, Chiba 277-8564 Japan

**Keywords:** Fabales, Genome size, Hairy root, *Polygala paniculata*, *rbc*L, RN symbiosis, Root hair response

## Abstract

Root nodule (RN) symbiosis is a mutualistic interaction observed between nitrogen-fixing soil bacteria and nodulating plants, which are scattered in only four orders of angiosperms called nitrogen-fixing clade. Most of legumes engage in RN symbiosis with rhizobia. Molecular genetic analyses with legumes and non-leguminous nodulating plants revealed that RN symbiosis utilizes early signalling components that are required for symbiosis with arbuscular mycorrhizal (AM) fungi. However detailed evolutionary processes are still largely unknown. Comparative analyses with non-nodulating species phylogenetically related to legumes could be better strategies to study the evolution of RN symbiosis in legumes. *Polygala paniculata* is a non-leguminous species that belongs to a family different from legumes but that is classified into the same order, Fabales. It has appropriate characteristics for cultivation in laboratories: small body size, high fertility and short lifecycles. Therefore, we further assessed whether this species is suitable as a model species for comparative studies with legumes. We first validated that the plant we obtained in Palau was truly *P. paniculata* by molecular phylogenetic analysis using *rbc*L sequences. The estimated genome size of this species was less than those of two model legumes, *Lotus japonicus* and *Medicago truncatula*. We determined conditions for cultivation in vitro and for hairy root formation from *P. paniculata* seedlings. It would facilitate to investigate gene functions in this species. The ability of *P. paniculata* to interact with AM fungi was confirmed by inoculation with *Rhizophagus irregularis*, suggesting the presence of early signalling factors that might be involved in RN symbiosis. Unexpectedly, branching of root hairs was observed when inoculated with *Mesorhizobium loti* broad host range strain NZP2037, indicating that *P. paniculata* has the biological potential to respond to rhizobia. We propose that *P. paniculata* is used as a model plant for the evolutionary study of RN symbiosis.

**Electronic supplementary material:**

The online version of this article (10.1007/s10265-019-01159-x) contains supplementary material, which is available to authorized users.

## Introduction

Symbiotic relationships between plants and soil microbes (fungi and bacteria) are a fundamental topic in biology. Symbioses between plants and mycorrhizal fungi are common among plant taxa; about 70% of flowering plant families form symbiotic relationships with arbuscular mycorrhizal (AM) fungi (Bonfante and Genre 2010; Brundrett 2009; Zhu et al. 2006). On the other hand, root nodule (RN) symbiosis with the nitrogen-fixing bacteria, rhizobia and *Frankia,* has been observed only in four orders (Fabales, Fagales, Cucurbitales and Rosales) within the subclade Fabids, which has been called the nitrogen-fixing clade (Doyle 2011; Griesmann et al. 2018; Kistner and Parniske 2002; Soltis et al. 1999; van Velzen et al. 2018, 2019). It has been predicted that genetic predisposition to RN symbiosis was acquired once in a common ancestor of these orders (Doyle 2011; Soltis et al. 1999; Werner et al. 2014). Phylogenomic analyses conducted in recent studies have shown that RN symbiosis was lost several times independently in ancestral lineages; therefore, RN symbiosis has been retained only in a few scattered members of the nitrogen-fixing clade (Griesmann et al. 2018; van Velzen et al. 2018). Two symbiotic systems originated in different eras; based on fossil evidence (Remy et al. 1994; Stubblefield et al. 1987) and molecular clock estimates (Simon et al. 1993), AM symbiosis originated over 400 million years ago (MYA), while the hypothesised predisposition to RN symbiosis first occurred ca. 100 MYA (Doyle 2011; Soltis et al. 1999). RN symbiosis is believed to have evolved through the recruitment of early signalling components of AM symbiosis (Kistner and Parniske 2002). However, details of the evolutionary process involved are still under investigation.

The Fabales, an order of Fabids, comprises four families: Fabaceae, Polygalaceae, Quillajaceae and Surianaceae (The Angiosperm Phylogeny Group 2016; Bello et al. 2009). Fabaceae is a family of leguminous plants species, about 90% of which engage in RN symbiosis with rhizobia. The other three families of the Fabales do not engage in this symbiosis (de Faria et al. 1989). The molecular basis of both RN and AM symbioses has been revealed using two model Fabaceae plants, *Lotus japonicus* and *Medicago truncatula* (Kouchi et al. 2010; Madsen et al. 2010; Oldrody 2013; Parniske 2008; Sato et al. 2008; Young et al. 2011). Comparative studies in Fabales between model legumes and closely related non-leguminous species belonging to the same order are useful for elucidating the evolutionary process of RN symbiosis. Herbs might be the most appropriate plant life form for laboratory-based experiments. The life forms of Polygalaceae include trees, shrubs and herbs, while Quillajaceae and Surianaceae are found only as trees (Stevens 2001). Thus, Polygalaceae species may be the most appropriate non-leguminous Fabales plants for comparative genomics and molecular genetic analysis to understand the evolution of the RN symbiotic system.

*Polygala paniculata* L. (subgenus *Polygala*, section *Timutua*) is an herbaceous plant belonging to the tribe Polygaleae and natively distributing in South America (Paiva 1998). This species has expanded its distribution as an introduced species in pan-tropical regions and some subtropical areas of Asia, such as the island of Taiwan and the Okinawa Islands (Yang and Chen 2013). Because this species has shown antifungal and analgesic activities in mice, local people in Brazil and areas to which it has been introduced have utilised this species to treat injuries, dislocations, etc. (Andrew et al. 2012; Frescura et al. 2012; Johann et al. 2011; Nogueira et al. 2005). The plant can grow to 10–50 cm tall and produces numerous racemes with over 100 flowers per inflorescence (Coelho et al. 2008). The flower is self-fertile and has a white, legume-like petal structure with two ovules per flower; one or two mature seeds can be obtained per fruit. The chromosome number of this species is reported as 2*n* = 52 or 56 (Favarger and Huynh 1965; Huynh 1965). A previous study mentioned the contents of only one germination medium for in vitro propagation (Nogueira et al. 2005) and there are no descriptions of the characteristics of *P. paniculata* as an experimental plant.

In the present paper, we characterised *P. paniculata*, a non-leguminous plant closely related to legumes, to assess its potential as a model plant for study of the evolution of plant–microbe interactions. To investigate the characteristics of this species as a model plant, we conducted molecular phylogenetic analysis and examined responses to AM fungal and rhizobial infections. We found that the estimated genome size of *P. paniculata* was similar to that of *L. japonicus* and *M. truncatula*. Additionally, a hairy root transformation method using *Agrobacterium rhizogenes* was established in *P. paniculata*. We introduce *P. paniculata* as a suitable experimental plant for reconstruction biology for the study of RN symbiosis.

## Materials and methods

### Plant materials

Seeds of *P. paniculata* used in this study were originally derived from the Republic of Palau (permission no. ROP–018-2014). Seeds were planted on vermiculite and cultured at 24 °C under a 16 h light and 8 h dark (16L: 8D) cycle in a growth chamber (Biotron, LPH-350S, NK system, Osaka, Japan). We harvested seeds from plants collected in Palau and used them for the following analyses.

### Phylogenetic analysis using chloroplast *rbc*L sequences

The genomic DNA of *P. paniculata* was extracted from the leaves of three individuals separately using a total protein solubilisation buffer [10 mM EDTA, 100 mM Tris–HCl buffer (pH 8.0), 1 M KCl] and chloroform (Wako, Japan) and resolved with Tris–EDTA (TE) buffer after ethanol precipitation. Primers used for both cloning and sequencing of the *P. paniculata rbc*L gene were designed with reference to the NCBI database and published papers on *Polygala* spp. (Bello et al. 2009; Fay et al. 1998; Forest et al. 2007; Käss and Wink 1996; Sulaiman et al. 2003). Then, *P. paniculata rbc*L DNA fragments were amplified with PrimeSTAR GXL DNA Polymerase (Takara, Japan) and the following primer set: 5′-ATGTCACCACAAACAGAAACTAAAGC-3′ and 5′-TATCCATTGCTTCGAAGACAAATTTG-3′ (Käss and Wink 1996). The DNA region amplified with these primers (1365 bp) included all the sequences of the *rbc*L gene for DNA barcoding (552 bp; CBOL Plant Working Group 2009). PCR was performed using an Applied Biosystems Veriti Thermal Cycler (Thermo Fisher Scientific, MA) with the following programme: 3 min at 98 °C, 30 cycles of 10 s at 98 °C, annealing at 55 °C for 15 s, 1 min at 68 °C a final extension of 7 min at 68 °C. After A-tailing with Ex-Taq DNA polymerase (Takara), PCR products were cloned into pGEM T-easy vector (Promega, Japan) and then insert DNA sequences were determined using Applied Biosystems 3130xl Genetic Analyzers (Thermo Fisher Scientific) with two sequencing primers specific to *rbc*L genes as follows: 5′-GCGTTGGAGAGACCGTTTCT-3′ (Fay et al. 1998) and 5′-TCGCATGTGCCCGCAGTAGC-3′ (Sulaiman et al. 2003).

Phylogenetic analysis of *P. paniculata* and other species belonging to Polygalaceae and Fabaceae was conducted. The nucleotide sequence data were searched using the keywords ‘Polygalaceae’ and ‘*rbc*L’ in the GENBANK database (accessed 30 March, 2017: NCBI Resource Coordinators 2016). Our search identified 408 sequences, from which we removed non-*rbc*L sequences and those that were too short for comparison. In total, 175 sequences, including those of our samples, were used for analyses (Table S1). DNA sequences were translated into amino acid sequences and multiple sequence alignment was conducted using the MAFFT programme (Katoh et al. 2019). Aligned sequences were then converted to nucleotide sequences again using PAL2NAL (Suyama et al. 2006). Because six of the sequences were in fact three pairs of identical sequences, only 172 sequences were used for the following analysis (Table S1). A maximum likelihood (ML) tree was constructed using RAxML version 8.2.6 with the GTRGAMMA model based on nucleotide sequences (Stamatakis 2014). Support values were calculated with 1000 bootstrap samples.

### Sterile culture conditions

Seeds were washed with 100% ethanol for a second and sterilised with 2–10% (w/w) hypochlorite solution (Wako) containing 0.2% Tween-20 (Amersham Biosciences, Sweden) for 40 min. The sterilised seeds were washed with sterilised water and planted on Murashige and Skoog (MS) medium [MS salt (4.6 g l^−1^), 0.2% (w/v) gellan gum (Wako), 0.1% (v/v) Gamborg’s vitamin solution (Sigma-Aldrich Co. LLC, MO), pH 5.8]. Sterilisation time, gellan gum concentration and sucrose concentration differed depending on the purpose of the experiment. Details of these differences are described in the result section separately. To determine the appropriate germination conditions, three replications were conducted for each treatment with ca. 30 seeds per plate. Plates were incubated at 24 °C under a 16L: 8D cycle.

### AM fungal inoculation

To confirm the AM fungal infection of *P. paniculata*, inoculation experiments were conducted using *Rhizophagus irregularis* (DAOM197198; Premier Tech, Canada) as previously described (Takeda et al. 2015). The inoculation pot consisted of three plastic parts: a lid, a soil container and a liquid medium reservoir. The soil was prepared by mixing vermiculite, gardening moulding and river sand in a ratio of 1:1:2 by volume, respectively and 100 ml one-tenth-strength Hoagland solution supplemented with 0.1 mM KNO_3_ (pH 5.8) was added. Extra medium fell into the reservoir from a pore at the bottom of the soil container. A Kimwipe (Crecia, Japan) was hung from the pore into the reservoir to transfer extra medium solution into the soil. *R. irregularis* spores (4000) were mixed in the soil. Four *P. paniculata* seedlings were planted in each pot with two replications and cultured in a growth chamber (24 °C, 16L: 8D) for 4 weeks. Roots were stained with ink as previously reported (Takeda et al. 2015) and fungal infection was observed under a stereomicroscope (SZX7, Olympus, Japan).

### Rhizobial inoculation

Seedlings of *P. paniculata* were grown on modified Broughton and Dilworth (B&D) medium (Broughton and Dilworth 1971) plates containing 0.5% sucrose, 0.6% gellan gum and 0.5 mM KNO_3_. To facilitate root hair formation, the concentration of KH_2_PO_4_ was reduced from 0.5 mM to 0.1 mM. Since B&D medium containing 0.6% gellan gum was hard to solidify, 0.5% plant agar was further added and the final concentration of MgSO_4_ 7H_2_O was adjusted to 1.5 mM. A bacterium with a broad host range, *Mesorhizobium loti* strain NZP2037 (Pankhurst et al. 1987), was used for the rhizobial infection experiment. Rhizobia were cultured in tryptone–yeast extract liquid medium at 28 °C for 2–3 d. The collected rhizobia were suspended in B&D medium at approximately 50-fold dilution, then spread on sucrose-free modified B&D plates. For the control of this experiment, B&D medium without rhizobia was spread on the plates. Seedlings with roots (1–3 cm in length) forming root hairs were transferred to these plates. Plates were stood and incubated at 24 °C under a 16L: 8D cycle. The area of the plates where roots elongated was shaded with black paper. Root hairs were observed 6–7 d after inoculation using a BX50 microscope (Olympus, Tokyo, Japan).

### Genome size estimation

Flow cytometric analysis was performed to estimate the size of the *P*. *paniculata* genome using CyFlow SL (Sysmex Partec, Japan). Three different organs (leaf, flower and inflorescence stem) from two different individuals were chopped up using a razor in nuclei extraction buffer (Sysmex Partec) and filtered through a 50 μm CellTrics filter (Sysmex Partec). Staining solution containing staining buffer, propidium iodide and RNase (Sysmex Partec) was added to samples. *Arabidopsis thaliana* leaves were used as an internal standard. The number of nuclei was measured using 488 nm blue lasers. A regression formula of genome size and peak intensities was constructed using *A. thaliana*, which has a genome size of 125 Mbp. Then, genome size of each *P. paniculata* sample was estimated from the regression formula.

### Hairy root transformation

Hairy root induction in *P. paniculata* seedlings was assessed using *A*. *rhizogenes* AR1193 transformed with a pCAMBIA1300GFP binary vector carrying the green fluorescent protein (*GFP*) gene driven by the cauliflower mosaic virus (*CaMV*) 35S promoter, which serves as a transformation marker for plant cells (Yano et al. 2017). Before transformation, *P. paniculata* seedlings were grown on MS medium (4.6 g l^−1^ MS salt, 0.2% gellan gum, 0.1% Gamborg’s vitamin solution, 0.1–1% sucrose, pH 5.8) under dark conditions for 1–2 weeks. *A. rhizogenes* was streaked on LB medium with 20 μg ml^−1^ kanamycin, incubated at 28 °C for 3 d and suspended in distilled water. *P. paniculata* seedlings were cut in the middle of the hypocotyls in the suspension to remove roots. Seedlings were incubated on co-culture medium (4.6 g l^−1^ MS salt, 0.2 g l^−1^ sucrose, 0.9% agar, pH 5.8) for 5–6 d in darkness then transplanted to root elongation medium [4.6 g l^−1^ MS salt, 1.0 g l^−1^ sucrose, 0.9% agar, 1 ml l^−1^ Gamborg’s vitamin solution, 12.5 mg l^−1^ meropen (Sumitomo Dainippon Pharm Co. Ltd., Japan), pH 5.8]. After 8 weeks of incubation, infections and hairy root generation were observed using a fluorescence stereomicroscope (SZX7, Olympus). Experiments were conducted three times under modified conditions, in the presence of 0.1 mM acetocyringone (AS) and/or 0.05 μM 1-naphthaleneacetic acid (NAA).

### Statistical analysis

Differences in genome sizes among organs were tested by the Kruskal–Wallis rank sum test. To test the effects of different treatments on germination efficiency and seedling viability, generalised linear model based analyses were conducted for each treatment. The explanatory variables of each treatment and link functions were set as the log of the binomial distribution. Significant differences in explanatory variables were tested by ANOVA and Tukey multiple tests were conducted for the significant explanatory variables in post hoc analysis. These analyses were performed in R software version 3.3.2. (R Core Team 2016). For comparative analyses of the number of deformed root hairs, significant differences were tested by Welch’s *t* test.

## Results

### Phylogenetic analysis

**Fig. 1:**
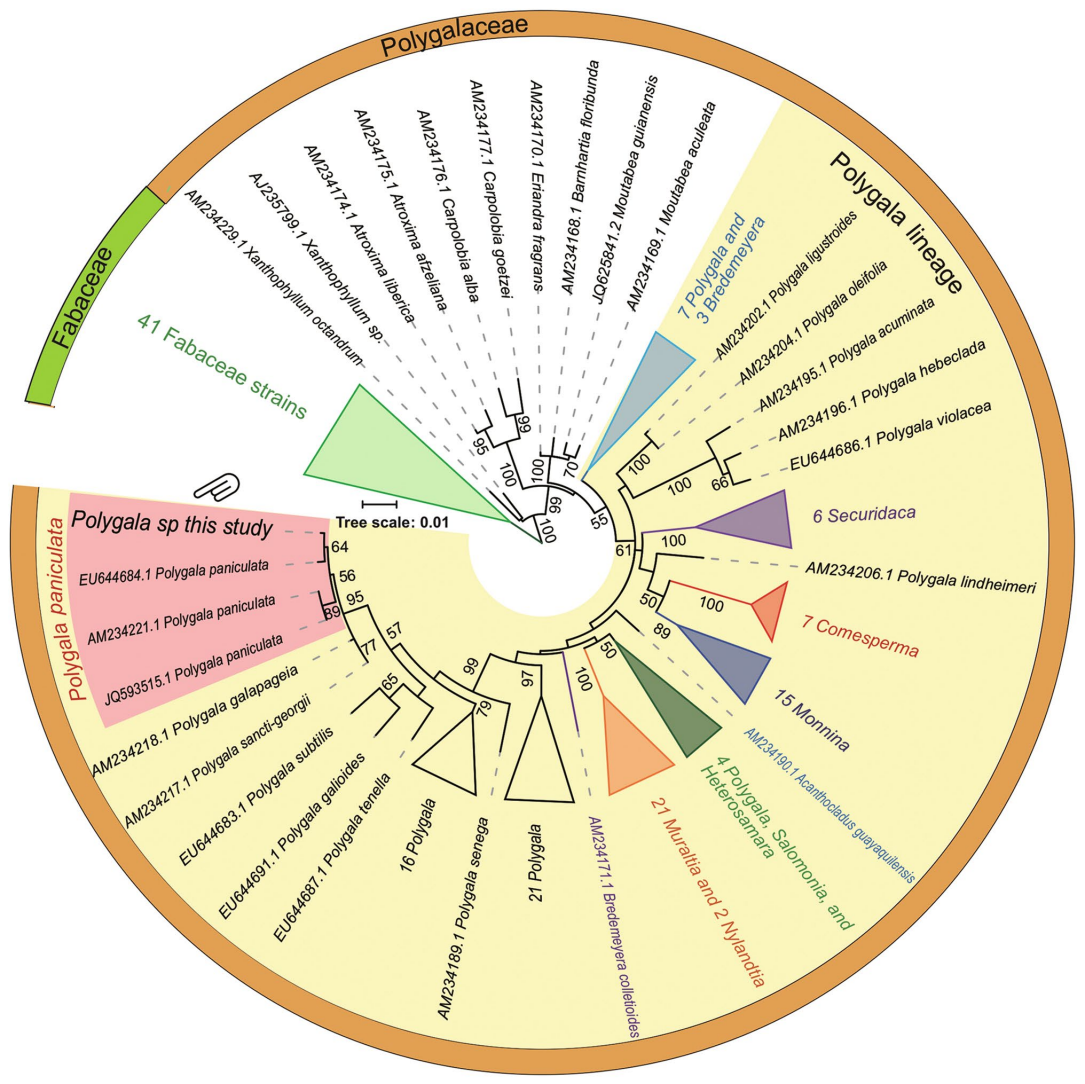
Schematic representation of the phylogenetic tree of the *rbc*L gene of Polygalaceae. The original phylogeny inferred by ML method using RAxML of DNA sequence evolution is shown in Fig. S1. The accession number and organism name of each gene were indicated on the leaf of the tree. The lineage of the genus *Polygala* and the species *P. paniculata* were respectively emphasized by yellow and red background colours. The leaves indicated with a hand arrow were of the *Polygala* plant used in this study. The lineage including the genus *Polygala* also includes nine other genera (highlighted with different colours). The number provided at a node corresponds with the bootstrap support greater than or equal to 50% from the ML analysis. The scale bar represents 0.01 substitutions per site

We investigated the phylogenetic relationships between the plants used in this study and Polygalaceae and Fabaceae species. We analysed the morphology and *rbc*L sequences of 172 plants, including three *P. paniculata* sequences from different individuals, and confirmed whether our sample was *P. paniculata* (Figs. 1, S1). The *Polygala* sample in this study and three *P. paniculata* sequences were monophyletic. Thus, the plant sample in this study was morphologically and molecularly confirmed as *P. paniculata*.

In the phylogenetic tree, interspecies relationships among Polygalaceae and Fabaceae plants were resolved. The results strongly support an interfamilial relationship between Fabaceae and Polygalaceae. Inter-tribe relationships among four Polygalaceae tribes were also strongly supported with high confidence values (bootstrap score: 99–100), except for the relationship between the tribes Polygaleae and Moutabeae (bootstrap score: 22, Table S1, Fig. S1). The genus *Polygala* was not monophyletic within the tribe Polygaleae; other genera, such as *Monnina*, *Securidaca* and *Comesperma* nested in *Polygala.* These results are supported by previous studies on phylogenetic relationships in Polygalaceae (Bello et al. 2009, 2012; Forest et al. 2007).

### Plant characteristics

**Fig. 2:**
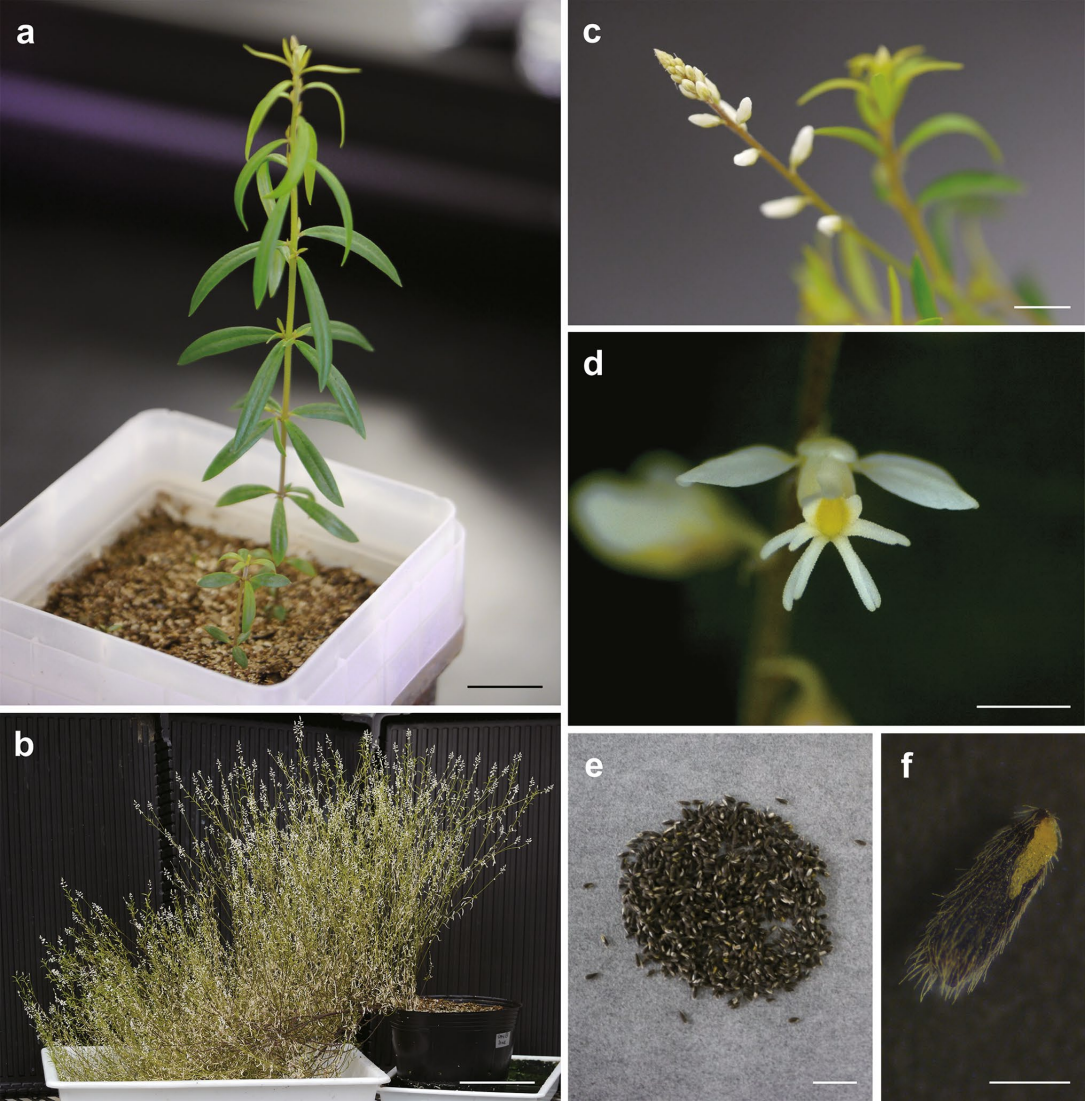
Representative images of *P. paniculata*. A seedling grown in a pot containing vermiculite and gardening mould for 6 weeks (**a**). A mature individual incubated under 8L: 16D conditions for 3 months after 16L: 8D conditions for 3 months (**b**). Inflorescence (**c**), flower (**d**), 1000 seeds (**e**) and a magnified image of a seed (**f**). Scale bars; 1 cm in (**a**, **c**), 10 cm in (**b**), 2 mm in (**d**), 5 mm in (**e**) and 0.5 mm in (**f**)

Plant characteristics and growth conditions in culture rooms or incubators constitute basic information used to deal with certain plants in laboratories. It was possible to cultivate *P. paniculata* in a plastic pot under our laboratory conditions (Fig. 2a), and the plants reached a final height of ca. 50 cm (Table 1, Fig. 2b, Paiva 1998). An initial inflorescence was observed 8 weeks after germination (Fig. 2c, d). *P. paniculata* is self-fertile and one or two seeds are produced from each flower. It took about 3 months to harvest new seeds. Over 5000 seeds were obtained from a mature plant with numerous inflorescences. The seeds were small (1.5 mm length) and had arilloids and dense hairs (Fig. 2e, f) that often repelled water. Plants were able to grow under both long-day (16L: 8D) and short-day (8L: 16D) conditions. Although long-day conditions were more effective for initial growth, the length of internodes gradually shortened and the plants could not produce mature seeds even after flowering. On the other hand, plants grown under 12L: 12D or 8L: 16D cycles continuously produced inflorescences. Therefore, for rapid growth and mass production of seeds, it would be better to culture plants under long-day conditions until initial inflorescence production, and then shift the light cycle to short-day conditions during seed harvesting periods.

**Table 1:** Summary of *Polygala paniculata* characteristics

Characteristics	References
Growth	
Small herb 10–50 cm	Paiva (1998)
Leaves alternate, but pseudowhorls at lowest 4–5 nodes	Coelho et al. (2008); Paiva (1998)
Period from seed to flowering, 8 weeks	This study
Generation time (seed to seed), 2–3 months	This study
Growth condition from germination to flowering, either 16L: 8D or 8L: 16D	This study
Growth conditions at seed maturation period, either 12L: 12D or 8L: 16D	This study
Reproduction	
Annual	Paiva (1998)
Self-fertile	This study
1 or 2 seeds per capsule	This study
More than 5000 seeds per plant	This study
Seed with densely hairs and arilloids	Paiva (1998)
1000 seed weight, 0.2188 g	This study
No seed scattering	This study
Genome	
2*n* = 52 or 56	Favarger and Huynh (1965); Huynh (1965)
Genome size, 403.5 Mbp C^−1^	This study
Hairy root	
Generated using *Agrobacterium rhizogenes*	This study

### Sterile culture conditions

Plant handling in vitro is a basic technique for transformation and experiments to assess plant interactions with microorganisms of interest. Although a previous study showed germination of *P. paniculata* on MS medium (Nogueira et al. 2005), the authors did not describe the detailed conditions for seed sterilisation and germination on the plate. We determined that the suitable condition for seeds sterilisation was 40 min incubation with a sterile solution of 2% sodium hypochlorite containing 0.2% Tween-20.

Next, we examined the concentrations of gellan gum and sucrose in the germination medium. Following the method detailed by Nogueira et al. (2005), we used MS medium supplemented with vitamins as the basic germination medium. Germinated seeds of *P. paniculata* sometimes displayed leaf yellowing or vitrification, and could not survive. Thus, we investigated not only germination rate but also their viability (Table 2). Although the germination efficiency did not differ among the four levels of gellan gum (*P* = 0.32, Table 2), viable seedling percentages increased with the amount of gellan gum (*P* < 0.01). The highest percentage of viable seedlings was observed on a 0.8% gellan gum plate [71.1 ± 6.1% (mean ± SE)]. However, the roots of the germinated seeds sometimes struggled to elongate into the medium, resulting in a growth delay. Leaves remained small and deposited anthocyanin as a stress response. Thus, the appropriate concentration of gellan gum was considered to be 0.6–0.8%. Like gellan gum, sucrose did not influence germination efficiency (*P* = 0.91, Table 2), but did increase the proportion of viable seedlings (*P* < 0.01). The three different sucrose concentrations (0.2–1.0%) conferred different viabilities on seedlings. Thus, the concentrations of gellan gum and sucrose critically influence the survival rate of seedlings immediately after germination. These two factors are very important in improving viability.

**Table 2:** Germination efficiency and seedling viability of *Polygala paniculata* on sterilised conditions

Experiment	Levels (%)	% of germinated seeds	*	% of viable seedlings^a^	*
Gellan gum concentration	0.2	80.5 ± 2.6	N.S.	3.4 ± 0.8	c
	0.4	65.33 ± 2.8	N.S.	13.3 ± 2.0	c
	0.6	78.06 ± 2.2	N.S.	45.3 ± 5.0	b
	0.8	80.72 ± 2.2	N.S.	71.1 ± 6.1	a
Sucrose concentration in 0.6% gellan gum plate	0.0	50.7 ± 0.8	N.S.	9.3 ± 0.8	c
	0.1	54.7 ± 2.8	N.S.	12.0 ± 2.7	bc
	0.2	54.7 ± 0.8	N.S.	34.7 ± 0.8	a
	0.4	50.7 ± 2.8	N.S.	28.0 ± 3.5	ab
	1.0	48.0 ± 4.8	N.S.	44.0 ± 4.8	a

Scores: mean ± SE *: ANOVA followed by Tukey multiple tests was used for statistical analysis. Different alphabets indicate different groups with significant differences (*P* < 0.05). N.S.: not significant. Seeds were sterilised with 10% sodium hypochlorite in this experiment

^a^Viable seedlings were seedlings that displayed neither leaf yellowing nor vitrification.  % viable seedlings were calculated from number of viable seedlings and number of germinated seeds

### Infection with AM fungi

**Fig. 3:**
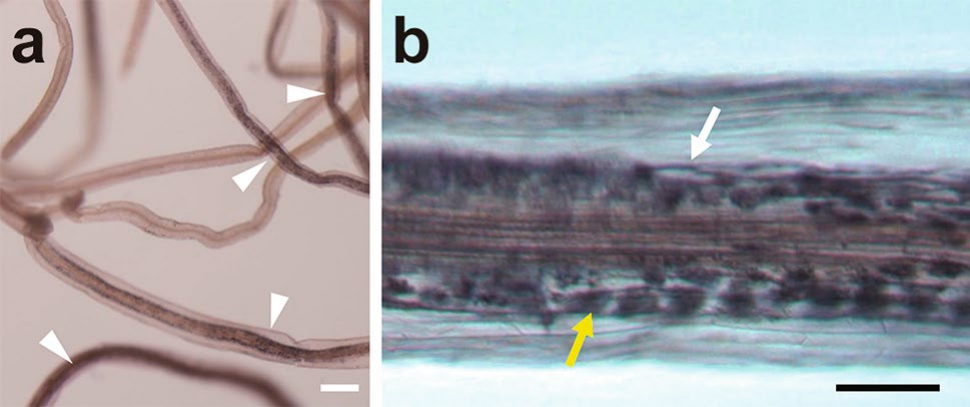
Infection in *R. irregularis* on *P. paniculata* roots. Roots at 4 weeks following inoculation were stained with black ink to visualise infection (**a**) and a magnified image of a root with arbuscules and hyphae (**b**). White arrowheads indicate infected roots. Yellow and white arrows indicate an arbuscule and a hypha, respectively. Scale bars; 0.5 mm in (**a**) and 0.1 mm in (**b**)

RN symbiosis evolved through the recruitment of early signalling components essential for AM symbiosis. Therefore, both symbioses share common factors involved in the regulation of early responses to microsymbionts (Kistner and Parniske 2002). To confirm the ability of *P. paniculata* to form a symbiotic relationship with AM fungi, 6-week-old plants were inoculated with *R. irregularis*. The roots were observed 4 weeks after inoculation. Although fungal infection did not fully expand to the entire root (Fig. 3a), all *P. paniculata* samples we observed were successfully infected by AM fungi (*N* = 4). Hyphae and arbuscules were developed in the cortex of infected roots (Fig. 3b). Thus, *P. paniculata* was able to be a host in AM symbiosis.

### Root hair responses to rhizobia

Although it is closely related to Fabaceae, *P. paniculata* is considered not to establish RN symbiosis with nitrogen-fixing bacteria. However, it may have partially retained the genes necessary for the symbiosis. Therefore, we examined whether *P. paniculata* responded to rhizobia. Since leguminous plants and rhizobia often show host specificity at the species level, we used a rhizobial strain with a broad host range, *M. loti* NZP2037, for inoculation (Pankhurst et al. 1987). This strain possesses additional *nod* genes not found in narrow host range *M. loti* strains (Kasai-Maita et al. 2013; Kelly et al. 2014).

**Fig. 4:**
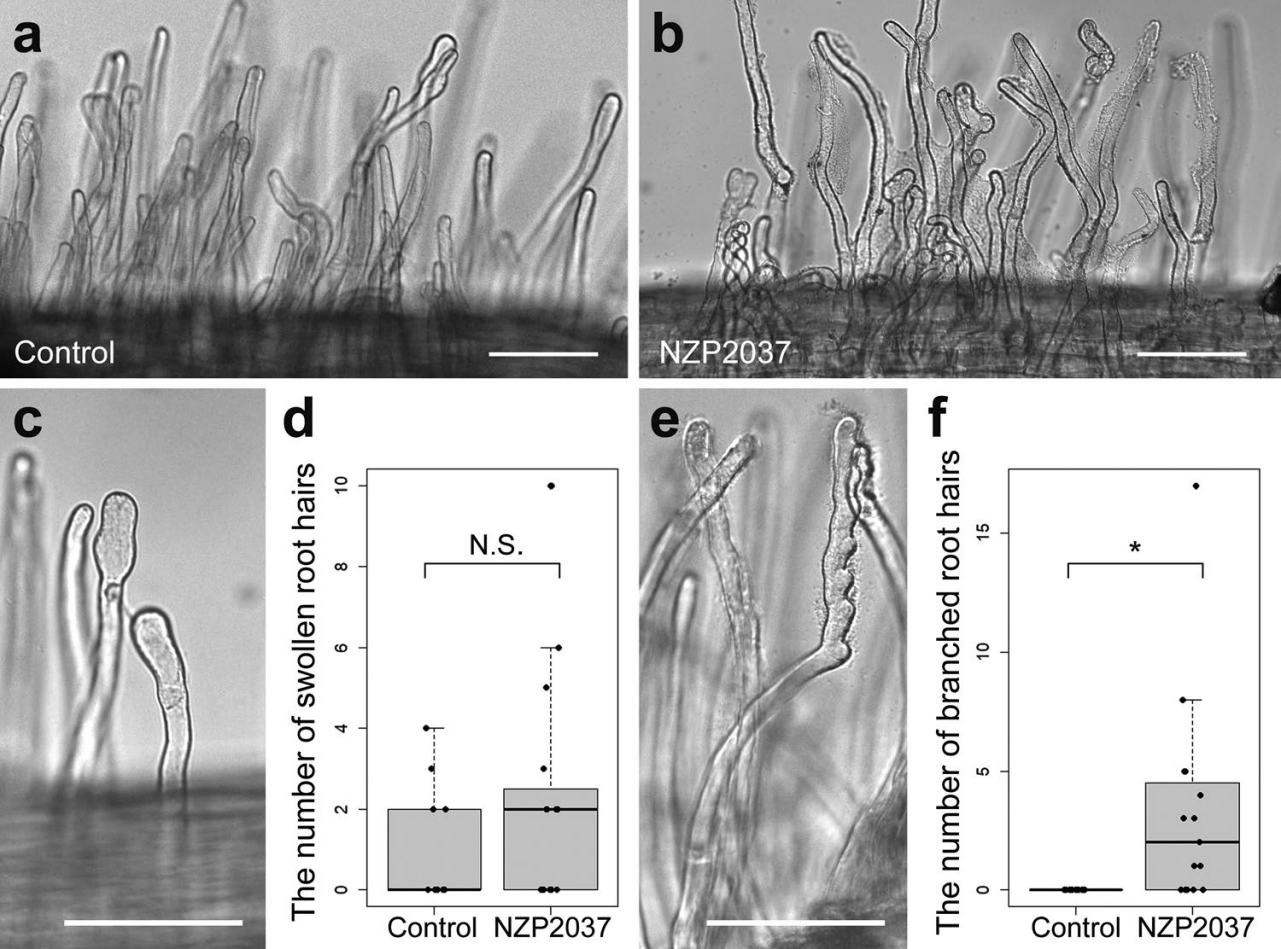
Root hair responses of *P. paniculata* to rhizobial inoculation. Broad views of root hairs in the control root (**a**) and the *M. loti* strain NZP2037-inoculated root (**b**). A typical image of swollen root hair tips (**c**) and the number of root hairs with swollen tips (**d**). A remarkable root hair branching observed in a *M. loti* NZP2037-inoculated root (**e**) and the number of branched root hairs (**f**). Statistical significance tests were performed using Welch’s *t* test (*N* ≥ 10). An asterisk indicates significant difference (*P* < 0.05). N.S.: not significant. Scale bars; 100 µm

Rhizobial inoculation is usually carried out under nitrogen starvation conditions to facilitate infection from root hairs and subsequent formation of RNs. When *P. paniculata* seeds were germinated on a nitrogen-deficient B&D agar medium, almost no root hairs were observed in the primary roots, at least in the early stages of seedling growth. To improve the productivity of root hairs, *P. paniculata* was grown on a B&D agar medium supplemented with 0.5% sucrose and a reduced amount of phosphorus. In this condition, ca. 80% of seedlings developed root hairs. Then, seedlings were transferred to a sucrose-free B&D agar medium and inoculated with rhizobia. There were no apparent differences in the growth and morphology of roots after inoculation with rhizobia when compared with non-inoculated roots. However, detailed observation revealed the occurrence of root hair deformation that was not seen in control plants (Fig. 4a, b).

The seedlings were inoculated with *M. loti* strain NZP2037 for 6–7 d to observe root hair responses. The tips of root hairs were occasionally observed to be swollen in both control and inoculated roots (Fig. 4c). There was no significant difference in the frequency of root hairs with tip swelling between control and inoculated roots (Fig. 4d). No clear root hair curling, which occurs to entrap rhizobia in leguminous roots, was observed in either control or inoculated roots (Fig. 4a, b). However, root hair branching was clearly observed only in roots inoculated with *M. loti* NZP2037 (Fig. 4b, e). The frequency of root hair branching in inoculated roots was significantly higher than that in control roots (Fig. 4f). This suggests that *P. paniculata* has the potential to recognise and respond to rhizobia.

### Genome size estimation

Genome size is an important factor that affects the efficiency of genetic studies and genome surveys. We performed flow cytometry to estimate the genome size of *P. paniculata* using three different organs (leaf, flower and inflorescence stem).

**Fig. 5:**
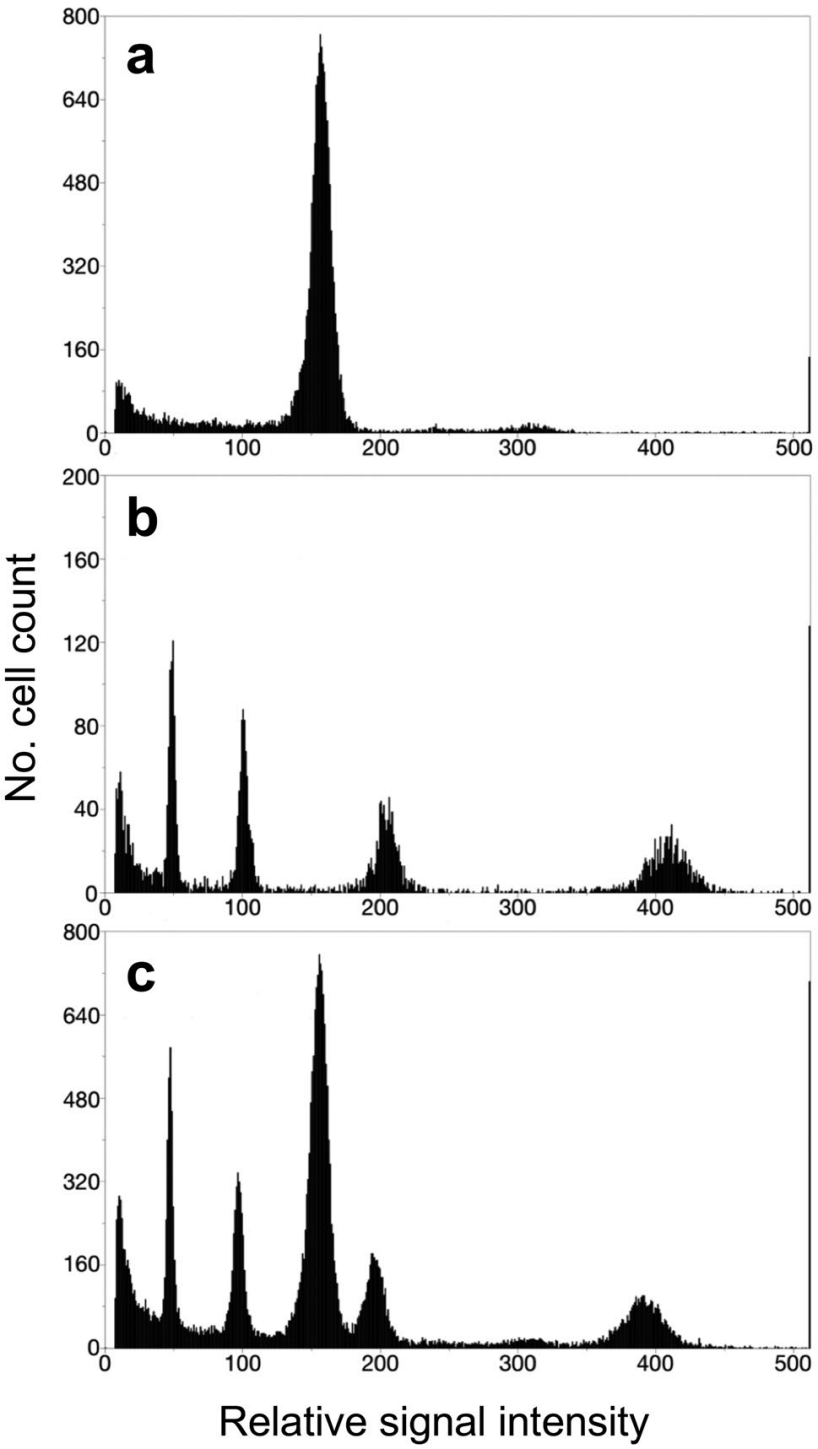
Flow cytometry to determine *P. paniculata* genome size. Histogram of relative signal intensities of the chromosome content per nucleus from *P. paniculata* sample (**a**), *A. thaliana* leaf (**b**) and their mixture sample (**c**)

Histograms of fluorescent intensities showed two peaks in leaf samples of *P. paniculata*: a large, sharp peak and a small, broad peak (Fig. 5a). The relative intensity of the small peak was approximately two-fold higher than that of the large peak, suggesting that the large peak represented nuclei with 2C and the small peak corresponded to 4C nuclei. *Arabidopsis thaliana* leaves had four peaks, with each peak representing each polyploidy (2, 4, 8 and 16C, Fig. 5b) and the regression formula had large R^2^ values ranging from 0.9996 to 0.9999. In mixed samples of *P. paniculata* and *A. thaliana* leaves, a peak corresponding to the 2C of the *P. paniculata* was positioned between the 4C and 8C peaks of *A. thaliana* (Fig. 5c). We repeated the same experiment using flowers and inflorescence stems of *P. paniculata*. Both samples had a single large peak corresponding to 2C. The estimated values slightly differed among *P. paniculata* organs, ranging from 389.4 Mbp C^−1^ in the inflorescence stem to 423.1 Mbp C^−1^ in the flower (Table 3); however, these differences in estimated genome size were not statistically significant (*χ*^2^ = 2.75, degree of freedom = 2, *P* = 0.25). The average genome size was 403.5 Mbp C^−1^ (*N* = 9, Table 3), which is comparable to those of *L. japonicus* and *M. truncatula*.

**Table 3:** *Polygala paniculata* genome size estimated by flow cytometry

Plant organ	Genome size (Mbp C^−1^)^a^	*N*
	Mean ± SE	
Leaf	398.1 ± 13.2	3
Flower	423.1 ± 8.0	3
Inflorescence stem	389.4 ± 13.9	3
Average	403.5 ± 8.9	9

^a^The size did not differ among organs (*P* = 0.25)

### Hairy root transformation of *P. paniculata*

Hairy root transformation is an efficient method to investigate gene functions in the root system. Therefore, we assessed hairy root transformation in *P. paniculata* seedlings. According to procedures for the hairy root transformation of *L. japonicus* (Okamoto et al. 2013), roots were cut off from the middle parts of etiolated seedlings that were cultured in darkness and shoots were inoculated with *A. rhizogenes* carrying a plasmid with the *GFP* gene driven by the *CaMV 35S* promoter as a transformation marker for roots.

**Fig. 6:**
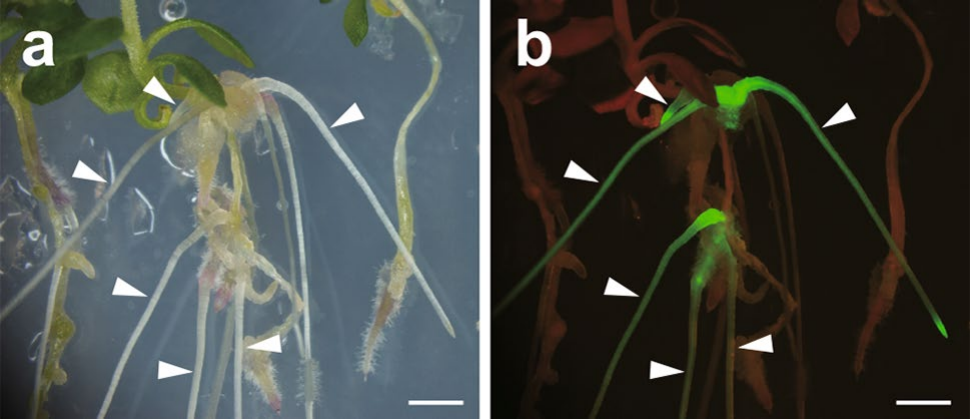
Hairy root transformation from a *P. paniculata* hypocotyl. A bright field (**a**), and its fluorescent image (**b**) depicting hairy roots. Arrowheads indicate hairy roots expressing GFP. Scale bars; 2 mm

We initially co-cultured *P. paniculata* shoots with *A. rhizogenes* on MS medium without AS for 5 d and then transferred the shoots to hairy root-inducing plates without any phytohormones to sterilise the bacterium strain and to induce hairy roots. Only one inoculated shoot in 53 individuals (1.9%) produced a gall-like tissue expressing GFP on the hypocotyl and hairy root induction was not observed (Table 4). To improve the low transformation efficiency, the co-culture medium was supplemented with AS. Additionally, NAA was added either into the hairy root-inducing plates or into both the co-culture medium and the hairy root-inducing plate to facilitate hairy root induction. The simultaneous treatment with AS and NAA increased the transformation efficiency of seedlings to 30.6% and stimulated hairy root generation in 17.5% of plants (Table 4; Fig. 6a, b). Hairy roots were successfully generated from *P. paniculata* hypocotyls using these procedures.

**Table 4:** The Summary of three hairy root induction tests of *Polygala paniculata*

Presence of AS in solution of *A. rhizogenes* suspension	Addition into co-culture medium	Addition into root elongation medium	No. individual	No. infected individual	No. infected individuals with hairy root	% of infection	% of hairy root induction
Absent	Absent	Absent	53	1	0	1.9	0.0
Present	AS	NAA	111	28	16	24.8	13.7
Present	AS, NAA	NAA	91	28	15	30.6	17.5

Concentration of AS: 0.1 mM in solution and medium, Concentration of NAA: 0.05 μM in medium

*AS* acetocyringone, *NAA* 1-naphthaleneacetic acid

## Discussion

### Characteristics of *P. paniculata*

Our research results provide fundamental knowledge about *P. paniculata* characteristics and suggest the possibility of further study using this plant species.

Analyses of phylogeny used 172 sequences obtained from the NCBI database, and relationships among the species in the family Polygalaceae were resolved. Polygalaceae comprises four tribes and ca. 900 species (Christenhusz and Byng 2016). The Polygalaceae plant species have diverged and some genera (*Bredemeyera*, *Muraltia*, *Nylandtia*, *Heterosamara*, *Salomonia*, *Acanthocladus*, *Monnina*, *Comesperma*, *Securidaca*, *Bredemeyera*) were nested in the genus *Polygala* (Figs. 1, S1). Similar results of the relationships among the *Polygala* clade and other genera are described in previous articles (Bello et al. 2009, 2012; Forest et al. 2007), indicating that our results based on *rbc*L analysis are reliable. To understand the evolution of legume–rhizobia symbiosis, knowledge of evolution of the four families within the order of Fabales was thought to be important (Doyle 2011). The evolution of the families has been in the middle of discussion and many studies strenuously analysed phylogenetic relationships among the families in this order (e.g., Banks et al. 2008; Bello et al. 2009, 2012; Forest et al. 2007; Qiu et al. 2010). These studies analysed several mitochondrial genes, such as *matK*, *matR*, *rbc*L, the *trnL* intron, *trnL*-*F* spacer, *atp1*, *nad5* and *rps3*. One of the studies which analysed *matK* and *rbc*L with several phylogenetic methods proposed that the families Polygalaceae, Fabaceae, Quillajaceae and Surianaceae arose under the orders listed above and that the Polygalaceae lineage diverged from other lineages of Fabales plants ca. 84 MYA (Bello et al. 2009). Doyle (2011) also postulated that Polygalaceae seems to have diverged ca. 80 MYA from other Fabales plants and that Fabaceae and Surianaceae arose ca. 10 million years later. Future studies using the whole genome information of *P. paniculata* and comparative studies with other Fabales plants including model legumes may resolve the phylogenetic relationships of the four families in Fabales.

Species in the genus *Polygala* are often associated with a wide taxonomical range of AM fungi within Glomeraceae 1 (GlGrA). Some genera in Polygalaceae, such as *Epirixanthes* spp., include species that exhibit mycoheterotrophy with AM fungi (Mennes et al. 2015; Rath et al. 2013, 2014). Thus, the relationship between *Polygala* and mycorrhizal fungi has diversified. We confirmed that *R. irregularis* successfully infected *P. paniculata* roots. Molecular genetic analyses of model legumes and non-leguminous plants have identified factors and signalling pathways involved in the symbiosis (Choi et al. 2018; Handa et al. 2015; Takeda et al. 2013). Comparative studies on the functional and molecular mechanisms of AM symbiosis between model legumes and *P. paniculata* may reveal differences in symbiotic mechanisms between Fabaceae and Polygalaceae.

The genome size of *P. paniculata* was estimated at 403.5 Mbp C^−1^, which is comparable with those of model plants in Fabaceae, *L. japonicus* (442 and 472 Mbp C^−1^) and *M. truncatula* (465 Mbp C^−1^) (Ito et al. 2000; Tang et al. 2014). Thus, the whole genome of *P. paniculata* could be analysed. Although the species diversity of the *Polygala* genus has been estimated (ca. 900 species), genome size has scarcely been determined (Castro et al. 2007; Coelho et al. 2008). According to a study on the genome sizes of two *Polygala* species by flow cytometry (Castro et al. 2007), one species *P. calcarea* belonging to subgenus *Polygala*, has relatively small genome size (481 Mbp C^−1^ in 34 chromosomes) and the other belonging to different subgenus, *P. vayredae* (subgenus *Chamaebuxus*), has relatively large size (1325 Mbp C^−1^ in 28 chromosomes). As well as the variety of genome sizes of plants of the genus *Polygala*, n-chromosome numbers also diverge greatly within the species of this genus (from 14 to 96 per 2*n*). Sharma and Mehra (1978) and Lack (1995) suggested that the burst of aneuploidy by hybridisation and expansion of their distribution cause the great inter-subgenus differences of n-chromosome numbers of *Polygala* spp. Lewis and Davis (1962) hypothesised that the basic number of chromosomes is 7, based on analysis of 52 *Polygala* spp. However, the critical data have not been shown until now. Analysing genome information of *P. paniculata* may clear inter-subgenus differences in genome size and chromosome number.

### Sterile culture and molecular genetic experiments

The germination method for other *Polygala* species were previously described as follows: seeds are embedded in agar for approximately 2 months after a short sterilisation (< 10 min) to absorb water and transplanted to germination medium for several further weeks (Royal Botanic Gardens Kew 2017). However, our germination protocol with longer period of sterilization treatment of seeds with sodium hypochlorite shortened the period necessary for *P. paniculata* seed germination. We also determined the appropriate concentrations of gellan gum and sucrose for *P. paniculata* viability were 0.6% and 0.2–1.0%, respectively. *P. paniculata* seedlings reached sizes enough for the other experiments, such as inoculation tests and hairy root transformation, within 2 weeks of germination under these conditions.

Furthermore, we examined the hairy root transformation in *P. paniculata* using *A. rhizogenes*. *L. japonicus* seedlings usually produce hairy roots in more than 50% of individuals under our experimental conditions (Okamoto et al. 2013). On the other hand, only one individual of *P. paniculata* seedlings (1.9% of all) produced gall-like tissues but did not induce any hairy roots on the same conditions (Table 4). Our analysis showed the importance of AS and NAA for the hairy root induction in *P. paniculata* seedlings. AS is one of the phenol-like products exuded from wounded plant organs. AS treatment often enhances the virulence of *Agrobacterium* spp., leading to the high transformation percentage in other plant species (Godwin et al. 1991; Kumar et al. 2006; Sheikholeslam and Weeks 1987). Requirement of AS for efficient *Agrobacteriun* infection varies according to plants (Godwin et al. 1991). *Nicotiana tabacum* shows that more than 20% of individuals are infected by *A. rhizogenes* without AS (Aoki and Syono 1999; Kumar et al. 2006). In the case of *P. paniculata,* we succeeded to increase frequencies of the infection and hairy root generation by simultaneous treatment of AS and NAA (Table 4); the infection rate and hairy root generation were 36.5% and 17.5% of *P. paniculata* seedlings. The successful induction of hairy roots would facilitate to analyse gene functions of interest and enable *P. paniculata* to be more useful as a model plant.

### Root hair responses to rhizobial infection

Leguminous plants evolved root hair infection to introduce rhizobia into host cells. The root hair infection requires root hair curling in response to the Nod factor secreted from rhizobia. Rhizobia are thereby entrapped in curled root hairs, allowing them to form an infection chamber and an infection thread (Oldrody 2013). In symbiotic mutants at the early stages of nodulation, curling of the root hairs does not occur and the root hair deformation such as branching, tip swelling, waving, etc. is frequently observed in an infection-dependent manner. We predicted that root hair deformation would not occur even when the non-leguminous plant *P. paniculata* was infected with broad host range rhizobia, but unexpectedly, clear branching was observed at a relatively high frequency only in roots infected with *M. loti* NZP2037 (Fig. 4e, f). This result indicates that *P. paniculata* has the potential to respond to rhizobia. In previous research, it has been shown that legume mutants deficient in the Nod factor signalling pathway such as *castor*, *ccamk* and *nup85* exhibit root hair branching in an infection- or Nod factor treatment-dependent manner (Imaizumi-Anraku et al. 2005; Miwa et al. 2006; Saito et al. 2007). When we infected plants with the AM fungus *R. irregularis* in the present study, arbuscules were formed (Fig. 3a, b). Thus, we predict that genes of the common symbiosis signalling pathway are maintained in the *P. paniculata* genome. On the other hand, in *M. truncatula*, root hair branching is induced in the symbiotic mutants of Nod factor receptors such as LYK3 (Smit et al. 2007). Therefore, in *P. paniculata*, it is predicted that mutations occur in RN-specific symbiotic genes. In the future, deciphering the genome of *P. paniculata* will define missing symbiotic genes and mutations at the microsynteny level.

In this paper, we proposed *P. paniculata* as a candidate experimental plant for the study of the evolution of RN symbiosis. *P. paniculata* is a species closely related to model legumes and it would be easier to conduct comparative genomics at microsynteny levels. This non-leguminous species has a small genome and a relatively short lifecycle. We showed that it can be cultured under sterile conditions in the laboratory. Hairy root transformation enables gene function analyses in *P. paniculata.* It is expected that *P. paniculata* will lead to better understanding of the evolution of RN symbiosis.

## Electronic supplementary material

Below is the link to the electronic supplementary material.

Supplementary material 1 (XLSX 46 kb)

Supplementary material 2 (PDF 2222 kb)

## References

[CR1] Andrew SM, Moe SR, Totland Ø (2012). Species composition and functional structure of herbaceous vegetation in a tropical wetland system. Biodivers Conserv.

[CR2] Aoki S, Syono K (1999). Synergistic function of *rolB, rolC*, ORF13 and ORF14 of TL-DNA of *Agrobacterium rhizogenes* in hairy root induction in *Nicotiana tabacum*. Plant Cell Physiol.

[CR3] Banks H, Klitgaard BB, Claxton F (2008). Pollen morphology of the family Polygalaceae (Fabales). Bot J Linn Soc.

[CR4] Bello MA, Bruneau A, Forest F (2009). Elusive relationships within order Fabales: phylogenetic analyses using *mat*K and *rbc*L sequence data. Syst Bot.

[CR5] Bello MA, Rudall PJ, Hawins JA (2012). Combined phylogenetic analyses reveal interfamilial relationships and patterns of floral evolution in the eudicot order Fabales. Cladistics.

[CR6] Bonfante P, Genre A (2010). Mechanisms underlying beneficial plant–fungus interactions in mycorrhizal symbiosis. Nat Commun.

[CR7] Broughton WJ, Dilworth MJ (1971). Control of leghemoglobin synthesis in Snake beans. Biochem J.

[CR8] Brundrett M (2009). Mycorrhizal associations and other means of nutrition of vascular plants: understanding the global diversity of host plants by resolving conflicting information and developing reliable means of diagnosis. Plant Soil.

[CR9] Castro S, Loureiro J, Rodriguez E (2007). Evaluation of polysomaty and estimation of genome size in *Polygala vayredae* and *P. calcarea* using flow cytometry. Plant Sci.

[CR10] CBOL Plant Working Group (2009). A DNA barcode for land plants. Proc Natl Acad Sci USA.

[CR11] Choi J, Summers W, Paszkowski U (2018). Mechanisms underlying establishment of arbuscular mycorrhizal symbioses. Annu Rev Phytopathol.

[CR12] Christenhusz MJM, Byng JW (2016). The number of known plants species in the world and its annual increase. Pytotaxa.

[CR13] Coelho VPM, Agra MF, Baracho GS (2008). Flora da Paraíba, Brasil: *Polygala* L. (Polygalaceae). Acta Bot Bras.

[CR15] de Faria SM, Lewis GP, Sprent JI (1989). Occurrence of nodulation in the Leguminosae. New Phytol.

[CR16] Doyle JJ (2011). Phylogenetic perspectives on the origins of nodulation. Mol Plant Microbe Int.

[CR17] Favarger C, Huynh KL (1965). *Polygala paniculata* L., 2n = 52–56. In Á. Löve (ed) IOPB IOPB chromosome number reports IV. Taxon.

[CR18] Fay MF, Bayer C, Alverson WS (1998). Plastid *rbc*L sequence data indicate a close affinity between *Diegodendron* and *Bixa*. Taxon.

[CR19] Forest F, Chase MW, Persson C (2007). The role of biotic and abiotic factors in evolution of ant dispersal in the milkwort family (Polygalaceae). Evolution.

[CR21] Frescura VD, Laughinghouse HD, do Canto-Dorow TS (2012). Pollen viability of *Polygala paniculata* L. (Polygalaceae) using different staining methods. Biocell.

[CR22] Godwin I, Todd G, Ford-Lloyd B (1991). The effects of acetosyringone and pH on *Agrobacterium*-mediated transformation vary according to plant species. Plant Cell Rep.

[CR23] Griesmann M, Chang Y, Liu X (2018). Phylogenomics reveals multiple losses of nitrogen-fixing root nodule symbiosis. Science.

[CR24] Handa Y, Nishide H, Takeda N (2015). RNA-seq transcriptional profiling of an arbuscular mycorrhiza provides insights into regulated and coordinated gene expression in *Lotus japonicus* and *Rhizophagus irregularis*. Plant Cell Physiol.

[CR26] Huynh KL (1965). Contribution à l’étude caryologique et embryologique des phanérogames du Pérou. Denkschriften der schweizerischen naturforschenden Gesellschaft.

[CR27] Imaizumi-Anraku H, Takeda N, Charpentier M (2005). Plastid proteins crucial for symbiotic fungal and bacterial entry into plant roots. Nature.

[CR28] Ito M, Miyamoto J, Mori Y (2000). Genome and chromosome dimensions of *Lotus japonicus*. J Plant Res.

[CR29] Johann S, Mendes BG, Missau FC (2011). Antifungal activity of five species of *Polygala*. Braz J Microbiol.

[CR31] Kasai-Maita H, Hirakawa H, Nakamura Y (2013). Commonalities and differences among symbiosis islands of three *Mesorhizobium loti* strains. Microbes Environ.

[CR32] Käss E, Wink M (1996). Molecular evolution of the Fabaceae: phylogeny of the three subfamilies based on *rbc*L-sequences. Biochem Syst Ecol.

[CR33] Katoh K, Rozewicki J, Yamada KD (2019). MAFFT online service: multiple sequence alignment, interactive sequence choice and visualization. Brief Bioinform.

[CR34] Kelly S, Sullivan J, Ronson C (2014). Genome sequence of the *Lotus* spp. microsymbiont *Mesorhizobium loti* strain NZP2037. Stand Genom Sci.

[CR35] Kistner C, Parniske M (2002). Evolution of signal transduction in intracellular symbiosis. Trends Plant Sci.

[CR37] Kouchi H, Imaizumi-Anraku H, Hayashi M (2010). How many Peas in a Pod? Legume genes responsible for mutualistic symbioses underground. Plant Cell Physiol.

[CR38] Kumar V, Sharma A, Narasimha Prasad BC (2006). *Agrobacterium rhizogenes* mediated genetic transformation resulting in hairy root formation is enhanced by ultrasonication and acetosyringone treatment. Electron J Biotechnol.

[CR39] Lack AJ (1995). Relationships and hybridization between British species of *Polygala* evidence from isozymes. New Phytol.

[CR40] Lewis WH, Davis SA (1962). Cytological observations of *Polygala* in eastern North America. Rhodora.

[CR41] Madsen LH, Tirichine L, Jurkiewicz A (2010). The molecular network governing nodule organogenesis and infection in the model legume *Lotus japonicus*. Nat Commun.

[CR42] Mennes CB, Moerland MS, Rath M (2015). Evolution of mycoheterotrophy in Polygalaceae: the case of *Epirixanthes*. Am J Bot.

[CR43] Miwa H, Sun J, Oldroyd GE (2006). Analysis of Nod-factor-induced calcium signaling in root hairs of symbiotically defective mutants of *Lotus japonicus*. Mol Plant Microbe Int.

[CR14] NCBI Resource Coordinators (2016) Database resources of the National Center for Biotechnology Information. Nucleic Acids Res 44 (D1):D7–D19. Accessed 30 March 2017. 10.1093/nar/gkv1290 PMC4702911 26615191

[CR44] Nogueira FLP, Fernandes SBO, Reis GM (2005). Atividade analgésica e antiedematogênica de *Polygala paniculata* L. (Polygalaceae) selvagem e obtida por micropropagação. Braz J Pharmacogn.

[CR45] Okamoto S, Yoro E, Suzaki T (2013). Hairy root transformation in *Lotus japonicus*. Bio Protoc.

[CR46] Oldrody GE (2013). Speak, friend, and enter: signalling systems that promote beneficial symbiotic associations in plants. Nat Rev Microbiol.

[CR47] Paiva JAR (1998). Polygalarum africanarum et madagascariensium prodromus atque gerontogaei generis Heterosamara Kuntze, a genere *Polygala* L. segregati et a nobis denuo recepti, synopsis monographica. Madrid: Cyanus. Fontqueria.

[CR48] Pankhurst CE, Hopcroft DH, Jones WT (1987). Comparative morphology and flavolan content of *Rhizobium loti* induced effective and ineffective root nodules on *Lotus* species, *Leuceana leucocephala, Carmichaelia flagelliformis, Ornithopus sativus,* and *Clianthus puniceus*. Can J Bot.

[CR49] Parniske M (2008). Arbuscular mycorrhiza: the mother of plant root endosymbiosis. Nat Rev Microbiol.

[CR50] Qiu YL, Li L, Wang B (2010). Angiosperm phylogeny inferred from sequences of four mitochondrial genes. J Syst Evol.

[CR70] R Core Team (2016) R: A language and environment for statistical computing. https://www.R-project.org/. Accessed 30 March 2017.

[CR51] Rath M, Weber HC, Imhof S (2013). Morpho-anatomical and molecular characterization of the mycorrhizas of European *Polygala* species. Plant Biol.

[CR52] Rath M, Grolig F, Haueisen J (2014). Combining microtomy and confocal laser scanning microscopy for structural analyses of plant-fungus associations. Mycorrhiza.

[CR53] Remy W, Taylor TN, Hass H (1994). Four hundred-million-year-old vesicular arbuscular mycorrhizae. Proc Natl Acad Sci USA.

[CR54] Royal Botanic Gardens Kew (2017) Seed information database (SID). Version 7.1. http://data.kew.org/sid/. Accessed 30 March 2017.

[CR55] Saito K, Yoshikawa M, Yano K (2007). NUCLEOPORIN85 is required for calcium spiking, fungal and bacterial symbioses, and seed production in *Lotus japonicus*. Plant Cell.

[CR56] Sato S, Nakamura Y, Kaneko T (2008). Genome structure of the legume, *Lotus japonicus*. DNA Res.

[CR57] Sharma ML, Mehra PN (1978). Chromosome numbers in some north west Indian species of *Polygala*. Cytologia.

[CR58] Sheikholeslam SN, Weeks DP (1987). Acetosyringone promotes high efficiency transformation of *Arabidopsis thaliana* explants by *Agrobacterium tumefaciens*. Plant Mol Biol.

[CR59] Simon L, Bousquet J, Lévesque RC (1993). Origin and diversification of endomycorrhizal fungi and coincidence with vascular land plants. Nature.

[CR60] Smit P, Limpens E, Geurts R (2007). Medicago LYK3, an entry receptor in rhizobial nodulation factor signaling. Plant Physiol.

[CR61] Soltis PS, Soltis DE, Chase MW (1999). Angiosperm phylogeny inferred from multiple genes as a tool for comparative biology. Nature.

[CR62] Stamatakis A (2014). RAxML version 8: a tool for phylogenetic analysis and post-analysis of large phylogenies. Bioinformatics.

[CR63] Stevens PF (2001) Angiosperm Phylogeny Website. Version 14. http://www.mobot.org/MOBOT/research/APweb/. Accessed 29 May 2019.

[CR64] Stubblefield SP, Taylor TN, Trappe JM (1987). Fossil mycorrhizae: a case for symbiosis. Science.

[CR65] Sulaiman SF, Culham A, Harborne JB (2003). Molecular phylogeny of Fabaceae based on *rbc*L sequence data: with special emphasis on the tribe Mimoseae (Mimosoideae). Asia Pac J Mol Biol Biotechnol.

[CR66] Suyama M, Torrents D, Bork P (2006). PAL2NAL: robust conversion of protein sequence alignments into the corresponding codon alignments. Nucleic Acids Res.

[CR67] Takeda N, Tsuzuki S, Suzaki T (2013). *CERBERUS* and *NSP1* of *Lotus japonicus* are common symbiosis genes that modulate arbuscular mycorrhiza development. Plant Cell Physiol.

[CR68] Takeda N, Handa Y, Tsuzuki S (2015). Gibberellins interfere with symbiosis signaling and gene expression and alter colonization by arbuscular mycorrhizal fungi in *Lotus japonicus*. Plant Physiol.

[CR69] Tang H, Krishnakumar V, Bidwell S (2014). An improved genome release (version Mt4.0) for the model legume *Medicago truncatula*. BMC Genom.

[CR71] The Angiosperm Phylogeny Group (2016). An update of the angiosperm phylogeny group classification for the orders and families of flowering plants: APG IV. Bot J Linn Soc.

[CR73] van Velzen R, Holmer R, Bu F (2018). Comparative genomics of the nonlegume *Parasponia* reveals insights into evolution of nitrogen-fixing rhizobium symbioses. Proc Natl Acad Sci USA.

[CR74] van Velzen R, Doyle JJ, Geurts R (2019). Resurrected scenario: single gain and massive loss of nitrogen-fixing nodulation. Trends Plant Sci.

[CR75] Werner GDA, Cornwell WK, Sprent JI (2014). A single evolutionary innovation drives the deep evolution of symbiotic N_2_-fixation in angiosperms. Nat Commun.

[CR76] Yang TYA, Chen CF (2013). A revision of the genus *Polygala* L. (Polygalaceae) in Taiwan. Taiwania.

[CR77] Yano K, Aoki S, Liu M (2017). Function and evolution of a *Lotus japonicus* AP2/ERF family transcription factor that is required for development of infection threads. DNA Res.

[CR78] Young ND, Debellé F, Oldroyd GE (2011). The *Medicago* genome provides insight into the evolution of rhizobial symbioses. Nature.

[CR79] Zhu H, Riely BK, Burns NJ (2006). Tracing nonlegume orthologs of legume genes required for nodulation and arbuscular mycorrhizal symbioses. Genetics.

