# Correction to: Assessment of *Polygala paniculata* (Polygalaceae) characteristics for evolutionary studies of legume–rhizobia symbiosis

**DOI:** 10.1007/s10265-021-01295-3

**Published:** 2021-04-20

**Authors:** Yuji Tokumoto, Kayo Hashimoto, Takashi Soyano, Seishiro Aoki, Wataru Iwasaki, Mai Fukuhara, Tomomi Nakagawa, Kazuhiko Saeki, Jun Yokoyama, Hironori Fujita, Masayoshi Kawaguchi

**Affiliations:** 1 Division of Symbiotic Systems, National Institute for Basic Biology, Okazaki, Aichi 444 8585 Japan; 2 Department of Basic Biology, School of Life Science, SOKENDAI (The Graduate University for Advanced Studies), Okazaki, Aichi 444 8585 Japan; 3 Department of Biological Sciences, Graduate School of Science, The University of Tokyo, Tokyo, 113 0032 Japan; 4 Division of Biological Science, Graduate School of Science, Nagoya University, Nagoya, Aichi 464 8602 Japan; 5 Department of Biological Sciences and Kyousei Science Center for Life and Nature, Nara Women’s University, Nara, 630 8506 Japan; 6 Faculty of Science, Yamagata University, Yamagata, 990 8560 Japan; 7 Astrobiology Center, Mitaka, Tokyo 181 8588 Japan; 8 Atmosphere and Ocean Research Institute, The University of Tokyo, Kashiwa, Chiba 277-8564 Japan

## Correction to: Journal of Plant Research (2020) 133:109–122 10.1007/s10265-019-01159-x

The article Assessment of *Polygala paniculata* (Polygalaceae) characteristics for evolutionary studies of legume–rhizobia symbiosis, written by Yuji Tokumoto, Kayo Hashimoto, Takashi Soyano, Seishiro Aoki, Wataru Iwasaki, Mai Fukuhara, Tomomi Nakagawa, Kazuhiko Saeki, Jun Yokoyama, Hironori Fujita and Masayoshi Kawaguchi, was originally published Online First without Open Access. After publication in volume 133, issue 1, page 109–122 the author decided to opt for Open Choice and to make the article an Open Access publication. Therefore, the copyright of the article has been changed to © The Author(s) 2021 and the article is forthwith distributed under the terms of the Creative Commons Attribution 4.0 International License (https://creativecommons.org/licenses/by/4.0/), which permits use, sharing, adaptation, distribution and reproduction in any medium or format, as long as you give appropriate credit to the original author(s) and the source, provide a link to the Creative Commons license, and indicate if changes were made.

The original article has been updated.

