# Supplementary material for: Assessment of Polygala paniculata (Polygalaceae) characteristics for evolutionary studies of legume–rhizobia symbiosis
Source: J Plant Res. 2019 Dec 11;133(1):109–22. doi: 10.1007/s10265-019-01159-x (PMC8057963; doi:10.1007/s10265-019-01159-x)
Supplement: Supplementary file 2 — Supplementary material 2 (PDF 2222 kb) [file 10265_2019_1159_MOESM2_ESM.pdf]

Electronic Supplementary Material (ESM) 2

Title: Assessment of *Polygala paniculata* (Polygalaceae) characteristics for evolutionary studies of legume–rhizobia symbiosis

Journal: Journal of Plant Research

Names of Authors: Yuji Tokumoto, Kayo Hashimoto, Takashi Soyano, Seishiro Aoki, Wataru Iwasaki, Mai Fukuhara, Tomomi Nakagawa, Kazuhiko Saeki, Jun Yokoyama, Hironori Fujita, Masayoshi Kawaguchi

Corresponding Author: Masayoshi Kawaguchi
National Institute for Basic Biology, Okazaki, Aichi, 444-8585 Japan
Mail: masayosi@nibb.ac.jp

Contents:

Fig. S1 Phylogeny of the *rbc*L gene of 172 sequences used in this work

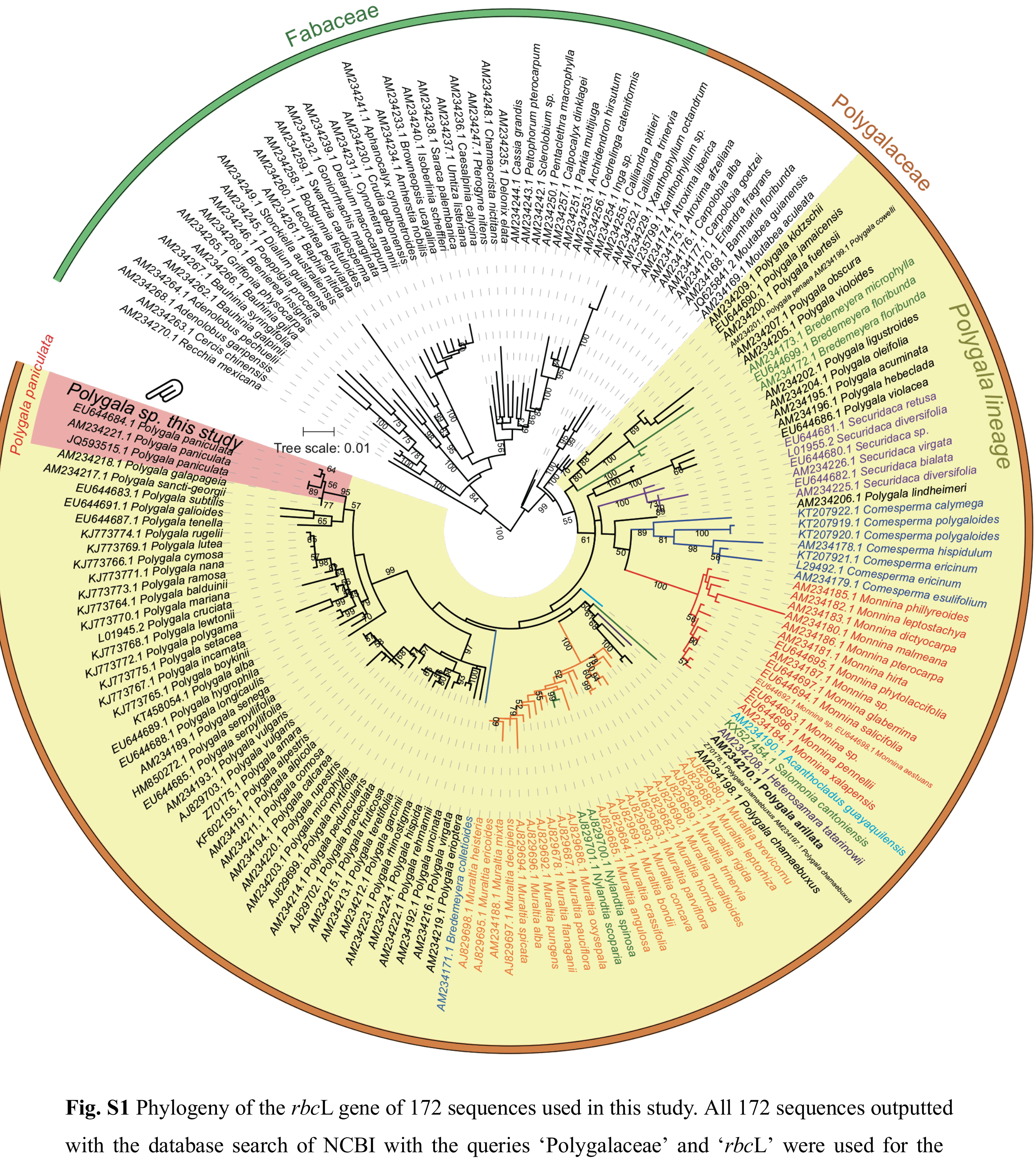

Fig. S1 Phylogeny of the *rbc*L gene of 172 sequences used in this study. All 172 sequences outputted with the database search of NCBI with the queries ‘Polygalaceae’ and ‘*rbc*L’ were used for the analysis. The accession number and organism name of each gene were indicated on the leaf of the tree. The lineage of the genus *Polygala* and the species *Polygala paniculata* were emphasised by surrounding yellow and red fan shapes, respectively. The leave indicated with a hand arrow was that of the *Polygala* plant used in this study. The lineage of the genus *Polygala* included nine other genera were coloured. The number provided at a node corresponds with the bootstrap support from the ML analysis. The scale bar represents 0.01 substitutions per site.
